# BAPTA, a calcium chelator, neuroprotects injured neurons in vitro and promotes motor recovery after spinal cord transection in vivo

**DOI:** 10.1111/cns.13651

**Published:** 2021-05-04

**Authors:** Kyu‐ree Kang, Jin Kim, Bokyeong Ryu, Seul‐Gi Lee, Min‐Seok Oh, Jieun Baek, Xiaoping Ren, Sergio Canavero, C‐Yoon Kim, Hyung Min Chung

**Affiliations:** ^1^ Department of Stem Cell Biology School of Medicine Konkuk University Seoul Korea; ^2^ Laboratory Animal Medicine College of Veterinary Medicine Seoul National University Seoul Korea; ^3^ Department of Orthopedics Ruikang Hospital Nanning China; ^4^ GICUP‐Global Initiative to Cure Paralysis Columbus Ohio USA; ^5^ HEAVEN/GEMINI International Collaborative Group Turin Italy; ^6^ Department of Physiology College of Veterinary Medicine Konkuk University Seoul Korea

**Keywords:** calcium, locomotor recovery, neuronal apoptosis, oxidative stress, spinal cord injury

## Abstract

**Aim:**

Despite animal evidence of a role of calcium in the pathogenesis of spinal cord injury, several studies conducted in the past found calcium blockade ineffective. However, those studies involved oral or parenteral administration of Ca++ antagonists. We hypothesized that Ca++ blockade might be effective with local/immediate application (LIA) at the time of neural injury.

**Methods:**

In this study, we assessed the effects of LIA of BAPTA (1,2‐bis (o‐aminophenoxy) ethane‐N, N, N′, N'‐tetraacetic acid), a cell‐permeable highly selective Ca++ chelator, after spinal cord transection (SCT) in mice over 4 weeks. Effects of BAPTA were assessed behaviorally and with immunohistochemistry. Concurrently, BAPTA was submitted for the first time to multimodality assessment in an in vitro model of neural damage as a possible spinal neuroprotectant.

**Results:**

We demonstrate that BAPTA alleviates neuronal apoptosis caused by physical damage by inhibition of neuronal apoptosis and reactive oxygen species (ROS) generation. This translates to enhanced preservation of electrophysiological function and superior behavioral recovery.

**Conclusion:**

This study shows for the first time that local/immediate application of Ca++ chelator BAPTA is strongly neuroprotective after severe spinal cord injury.

## Graphical abstract

BAPTA, a cell‐permeable selective calcium chelator which showed the effect in several neuronal disease models, was assessed in spinal cord injury mice model and in vitro model of neuronal damage. BAPTA improved behavior recovery by reducing neuronal death in vivo model. Also, BAPTA help to decrease neuronal death and relieve the impairment of electrophysiological function on in vitro model.

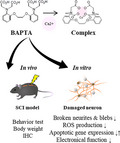

## 1 INTRODUCTION

Spinal cord injury (SCI) can be due to trauma (84%) or non‐traumatic causes (16%), with about 17 000 new SCI cases each year in the United States alone.^1^ SCI usually induces irreversible dysfunction of the motor, autonomic, and sensory systems,^2^ for which no clinically available treatment is available.

Traumatic spinal cord injury (SCI) is associated with immediate mechanical disruption of neurons, fibers, glia, and vessels (primary injury). Thereafter, an acute phase *secondary injury cascade* sets in within minutes to hours. During this phase, injured glial and nerve cells in the spinal cord undergo cell death, compounded by disruption of blood vessels and *ischemia*. Vessel disruption is accompanied by accumulation of inflammatory cells at the site of injury, with ensuing spinal cord swelling, causing further spinal cord compression, and worsening.^3^, ^4^, ^5^ In the subacute phase (2‐14 days), an imbalance of cell homeostasis, cell death, and inflammatory cellular responses set in. The secondary injury cascade may prove more damaging than the original trauma.^6^ Calcium dysregulation is a key step in this pathophysiological cascade.^7^, ^8^, ^9^, ^10^, ^11^, ^12^ In particular, traumatic white matter injury appears to be mediated both by ryanodine‐sensitive receptors (RyRs) and through inositol (1,4,5)‐triphosphate receptor (IP3Rs) Ca++induced Ca++ release receptors (CICRs).^13^ However, calcium channel blockade has not been proven neuroprotective in clinical SCI trials.^14^

We hypothesized that Ca++ blockade might be more effective if applied locally and at the time of injury. Although this application may be difficult in the clinic, still this could provide valuable data for future drug development. BAPTA(1,2‐bis (o‐aminophenoxy) ethane‐N, N, N′, N'‐tetraacetic acid), a cell‐permeable selective calcium chelator, buffers excessive calcium influx and mitigates neural injury, for example, in ALS models^15^ or following cortical stab injury.^16^

The aim of the present study was thus two fold:

1. Assessing for the first time whether locally administered BAPTA can promote behavioral recovery after spinal cord transection in mice and concurrently evaluate associated histological changes at the site of injury;
2. Assessing in vitro possible mecshanisms of neuroprotection by employing neuron‐differentiated neural stem cells (NSC) as a surrogate of locally injured gray matter spinal neurons.

## 2 MATERIAL AND METHODS

### 2.1 In vivo assessment

#### 2.1.1 Animal used and ethics

All experiments described adhered to the rules developed under the United States Department of Agriculture's (USDA) Animal Welfare Act and adopted ARRIVE instructions.^17^ Also, the study was approved by the Konkuk University IACUC (approval number KU15135). A total of 26 mice were used in the experiment. Spinal cord transection was performed on 23 animals excluding the sham group, and 3 of them were euthanized appropriately due to severe weight loss after surgery, so they were excluded from the experiment. The number of animals per group was determined considering previous studies.^18^, ^19^, ^20^, ^21^, ^22^ Finally, twelve of the 23 mice were used as the BAPTA group, 7 as the vehicle control group, and 3 as the sham group.

#### 2.1.2 Spinal cord transection

Six‐week‐old BALB/c female mice were obtained from Orient Bio Inc., Republic of Korea. The mice were housed in groups of five in cages under 12 hours light/dark cycle with water and standard rodent chow provided *ad libitum*. Mice were anesthetized using Zoletil (1:1 composite of tiletamine and zolazepam) and xylazine (3:1 ratio, 1 mL/kg). Spinal laminectomy was performed at the T9 level. To expose the spinal cord, the muscles overlying the vertebral column were cut and the dura was incised with surgical scissors. The hook was passed ventrally the spinal cord and carefully lifted to ensure the entire spinal cord was present in hook's bent. Afterward, the full spinal cord was incised by a surgical blade. The gauze was put on the incised spot for 5 seconds to drain and removed with a hook. For treatment, 20 μL of 10 mM BAPTA (Sigma, USA) diluted in DPBS (Welgene, Republic of Korea) and DPBS were prepared. The concentration of BAPTA was determined considering previous studies.^23^, ^24^, ^25^ The BAPTA or DPBS were treated in drops on the full‐transection spot with pipette and were arranged into BAPTA group and vehicle control group, respectively. The muscle and fascia were sutured, and the skin was closed. Following the surgery, additional water and crushed food were provided for the first 14 days after SCI. Mice had their bladders expressed manually three times a day. Animals in a sham group were subjected to the same surgical procedures except that spinal cords were not injured.

#### 2.1.3 Histology and Terminal deoxynucleotidyl transferase dUTP nick end labeling (TUNEL) assay

For histological analysis, the sections were stained with TUNEL assay. All 23 mice were sacrificed at day 28 post‐injury. The spinal cord was isolated and fixed with 4% paraformaldehyde (Biosesang, Republic of Korea) for five days. Tissue samples were embedded in paraffin, and transverse sections (10 μm thick) were mounted on slides. The spinal cords from 10 mice in BAPTA group and 5 mice in vehicle control group were used for histology. The spinal cords from 2 mice in sham group were also used. For quantification of apoptotic cells, the double staining of TUNEL and NeuN (1:100, ab128886, Rabbit) was conducted. The TUNEL assay (Thermo Fisher, USA) was performed according to the manufacturer's instruction (Thermo Fisher) and described previously.^26^ Images from different slides were captured by florescence microscope, Nikon TE2000‐U (Nikon; Japan). The percentage of TUNEL‐positive neurons relative to the total number of cells labeled with Hoechst was calculated with ImageJ software (National Institutes of Health and the Laboratory).

#### 2.1.4 Modified Basso Beattie Bresnahan (mBBB) score

The modified Basso Beattie Bresnahan locomotor rating scale was used to evaluate behavioral recovery in an open field. mBBB score was measured following original study.^27^ A total of 12 mice were included in the BAPTA group, and another 7 mice were included in vehicle control group. Sham group involved 3 mice. Two independent, blinded observers evaluated the spinal cord sensory and motor function of the animals after spinal cord transection. Behavioral recovery was tested every three days for four weeks.

### 2.2 In vitro assessment

#### 2.2.1 Isolation of neural stem cells

Neural stem cells (NSCs) were isolated from C57BL/6 mouse brains at embryonic day 14. The forebrain was separated and dissected out and digested in 0.25% trypsin‐EDTA (Thermo Fisher) for 10 minutes at 37°C. Dissected tissues were resuspended briefly by pipetting and filtering them through a 40‐μm strainer after inactivation of trypsin‐EDTA by FBS (Thermo Fisher). Cell suspensions were plated on Matrigel (Thermo Fisher)‐coated dishes in an NSC culture medium (DMEM/F12 [Thermo Fisher]) supplement with 1% N2 (Thermo Fisher), 2% B27 (Thermo Fisher), 1% penicillin/streptomycin (Thermo Fisher), 1% GlutaMAX (Thermo Fisher), and 10 ng/mL of bFGF and EGF (PeproTech, USA). Before using them in experiments, NSCs were processed multiple times (>3).

#### 2.2.2 In vitro differentiation of NSCs into neurons

For all of in vitro assessments, mouse neuron was differentiated from NSCs. The NSCs were seeded at 2 X 10^5^ cells on poly‐L‐lysine (Sigma)/laminin (Thermo Fisher)‐coated 6‐well plates. The next day, the cells were cultured in a differentiation medium 1: DMFM/F‐12 supplemented with 1% N2, 2% B27, 1% penicillin/streptomycin, 1% glutamine, and 10 ng/mL of bFGF. On day seven of differentiation, the medium was changed to a differentiation medium 1 without bFGF for 14 more days. The culture medium was refreshed every day. Differentiated neurons showed typical neuron morphology such as a center‐located cell body with branching neurites having long and thin tunnel structures (Figure S1A). Neurons also expressed typical neuronal markers such as neurofilament1 (NF1) (Figure S1B). Furthermore, an RT‐PCR demonstrated fully differentiated neurons not expressing astrocytes, oligodendrocytes, or NSC genes (Figure S1C and Figure S3A).

#### 2.2.3 Impairment method by glass bead

To give physical damage on neuron, a 425‐600 μM diameter glass bead was used. Also, in order to prevent the cells from being damaged due to the difference in ion concentration and osmotic pressure, a buffer solution DPBS which have the same ion concentration and osmotic pressure as in vivo was used. Considering that calcium chloride and magnesium contained in DPBS may affect BAPTA action, a DPBS from which calcium chloride and magnesium chloride were removed was adopted. For physically damaging on neuron, 100 mg of glass beads (Sigma) were mixed with differentiated neurons sunk with DPBS. The plates were gently rocked three times to let the beads roll over the cells following previous study.^28^ DPBS and the glass beads were removed by suction. Before the experiment, BAPTA was prepared by diluting in DMEM/F12 at final concentrations of 10 μM, 20 μM, and 40 μM. Each of the prepared BAPTA (BAPTA group) or DPBS (vehicle control group) was gently treated on the cell. Then, the plate was incubated in 37°C for 30 minutes so that BAPTA could protect the damaged cells. During this process, the sham group was not damaged and no specific substances were treated. Cells were detached by treatment with TrypLE (Thermo Fisher) and incubating the plate at 37°C for 5 minutes. Finally, the cells were collected by DMEM/F12 and used for in vitro experiments.

#### 2.2.4 Cell mortality using trypan blue

To evaluate the effect of BAPTA on cell death, the mortality of cells was quantified by trypan blue staining. First of all, the differentiated neurons were physically damaged by glass beads as described in section 2.2.3. Then, the plates were incubated in BAPTA or DPBS (vehicle control) for 30 minutes at 37°C. Right after finishing BAPTA treatment, the culture supernatant was collected for measurement of cell mortality. In case of sham group (no damage, no treatment), they were incubated in a 37°C, 5% CO_2_ incubator until supernatant collection. Each of the collected culture supernatant was spun down at 300G for 5 minutes and reconstituted with 1 mL of empty media. 10 μL of the resuspended sample was mixed with 10 μL of 0.4% trypan blue (Thermo Fisher) by gentle pipetting. Then, 20 μL of the mixture was loaded onto each side of the hemocytometer. The blue staining cells placed into the four corner quadrants were counted, and an average taken by multiplying them by 2 × 10^4^. Afterward, TrypLE™ was treated for 5 minutes and incubated at 37°C to detach all cells, and those live cells which were not stained by trypan blue were counted in the same way. The total number of cells was calculated by plus the number of blue staining cells (dead cells) and unstained cells (live cells). Finally, the percentage of dead cells was obtained by dividing the number of living cells by the total number of cells. Counts were done in triplicate under 40x magnification.

#### 2.2.5 Measurement of the reactive oxygen species (ROS) level

To evaluate the effect of BAPTA on ROS production, the level of ROS was measured by DCF‐DA assay. Firstly, a different batch of differentiated neurons was detached immediately with TrypLE™ (Thermo Fisher) and seeded onto a 96‐well plate at 200 cells/ well. Different concentrations (10 μM, 20 μM, and 40 μM) of BAPTA were applied on the cells and incubated for 30 minutes at 37°C. To measure the ROS level, a 2′,7′‐dichlorodihydrofluorescein diacetate (DCF‐DA) assay kit (Thermo Fisher) was loaded and incubated for 30 minutes. All fluorescence signals were measured in quantitative values by using a fluorescence microplate reader (Gemini EM, Molecular Devices, USA) with excitation and emission wavelengths of 485 and 535 nm, respectively.

#### 2.2.6 Scanning Electron Microscopes (SEM) imaging

To confirm the ability of BAPTA to mitigate physical damage of neurons, images of each groups were taken using SEM. Samples were fixed using 2% glutaraldehyde (Sigma), 2% paraformaldehyde (Sigma) in 0.05 M sodium cacodylate buffer for 4 hours at room temperature and washed three times for 5 minutes in 0.05 M of sodium cacodylate buffer. For secondary fixation, we placed 2% osmium tetroxide in 0.1 M of sodium cacodylate buffer for 1 hour at 4°C. Then, samples were rinsed several times with distilled water and dehydrated completely with 30%, 50%, 70%, 80%, 90%, and 100% ethanol. After treatment of hexamethyldisilazane (HMDS) for 20 minutes at room temperature, the dry CO_2_ at supercritical point was used to remove any residual solvents. Finally, a thin layer of Au covered the samples. SEM analyses and photographs were taken with an AURIGA field‐emission scanning electron microscope (ZEISS, Germany).

#### 2.2.7 Quantitative Real‐time PCR (qPCR)

To confirm the effect of BAPTA on apoptosis related gene expression, 40 μM of BAPTA were applied for 30 minutes after bead rolling. After 12 hours, damaged neurons were collected for the qPCR. GAPDH gene was used as an internal standard. To extract the RNA, trizol reagent (Thermo Fisher) was used. Total RNA was reverse‐transcribed into complementary DNA (cDNA) using the cDNA synthesis kit (Thermo Fisher) following the manufacture's protocol. PCR reaction was carried out using the SYBR® Green Quantitative RT‐qPCR Kit (Roche, Switzerland) and a Roche real‐time PCR system (LightCycler® 96). ΔCt values were calculated by subtracting the GAPDH Ct value from that of target genes. Relative expression levels were calculated using the 2^−ΔΔCt^ method. Primer sequences and the size of amplicons are listed in Table 1.

**TABLE 1:** Primer list

Gene name	Band size (bp)	Sequence		Annealing temperature (°C)
Mouse caspase 3	254	Forward	TCTGGTACGGATGTGGACGC	61.94
		Reverse	CGGCAGTAGTCGCCTCTGAA	61.93
Mouse Caspase 9	111	Forward	ATGGAGATGGCACACCGGAA	61.56
		Reverse	TGTCCCATAGACAGCACCCG	61.61
Mouse Bcl‐2	254	Forward	CGTTGGCCCTTCGGAGTTTA	60.32
		Reverse	TATCCACCGGACCGCTTCA	60.68
Mouse Bcl2 l1	111	Forward	AGGCAGGCGACGAGTTTGAA	62.38
		Reverse	AAGCTGCGATCCGACTCACC	62.56

#### 2.2.8 Immunocytochemistry

The cells were fixed with 4% paraformaldehyde (Biosesang, Republic of Korea) for 30 minutes at room temperature and washed three times with DPBS. After that, blocking solution containing 0.1% Triton X‐100 (Promega, USA) and 5% FBS (Thermo Fisher) diluted with DPBS were treated for one hour at room temperature. The cells were incubated with primary antibodies, NF1(Abcam, UK) at 4°C for 16 hours, washed three times with DPBS, and finally incubated with appropriate fluorescence‐conjugated secondary antibodies, Alexa Fluor 594 goat anti‐Rabbit IgG (Thermo Fisher), for 2 hours at room temperature without light. Nuclei were stained with Hoechst 33342 (Thermo Fisher).

#### 2.2.9 Terminal deoxynucleotidyl transferase dUTP nick end labeling (TUNEL) assay

The TUNEL assay (Thermo Fisher) was performed to evaluate neuronal apoptosis. NSCs were seeded on a coverslip and differentiated into neurons as described above. After damaging by glass beads, different concentrations of BAPTA were treated for 30 minutes at 37°C. After that, neurons were fixed with 4% paraformaldehyde in DPBS followed by permeabilization with 0.25% Triton X‐100. Images from three different fields, containing about 250 cells in three independent experiments, were captured. The percentage of TUNEL‐positive neurons relative to the total number of cells labeled with Hoechst was calculated with ImageJ software (National Institutes of Health and the Laboratory).

#### 2.2.10 Multiple‐electrode array (MEA) measurement

To confirm that BAPTA treatment can protect neuronal function by relieving the electrophysiological damage of neurons, BAPTA (final 10 μM) was applied to neuron scratched by glass hook for 30 minutes.

Then, the mean firing rate (MFR) of neurons was measured by MEA every 5 days to evaluate the electrophysiological properties. The MEA plate was maintained in the incubation at 37°C and with 5% CO_2_. Every 5 days, the whole media were changed and the MEA plate was placed in a 37°C, 5% CO_2_ incubator for 1 hour to an adaptation of cells to the new media. After then, the MEA plate was put into axion maestro (Axion BioSystem Inc, USA) conditioned at 37°C and 5% CO_2_ conditions for measuring MFR. Because the spontaneous electrophysiology is very sensitive to CO_2_ concentration, the MEA plate placed in axion maestro for 15 minutes for stabilization, and after that the MFR was measured. Data recording and acquisition were managed with Axion's Integrated Studio. The gain of the amplifier was 1200, and the input voltage range of the data acquisition board was set from –819 to +819 mV. Data were sampled at a frequency of 25 kHz/channel with a 200 Hz band‐pass filter.

#### 2.2.11 Analysis of MEA data

The saved data files were imported into MS Excel for the calculation of the mean firing rate (MFR). For MFR determinations, the final 10‐minute recordings of the whole recording file were considered as baseline. A channel is defined as active if it exhibits 10 or more spikes/minute during the reference phase of activity. For MFR, only the channels that were active (=/>10 spikes/min) during the reference phase of the recordings were selected for analysis.

#### 2.2.12 Statistical analysis

**FIGURE 1:**
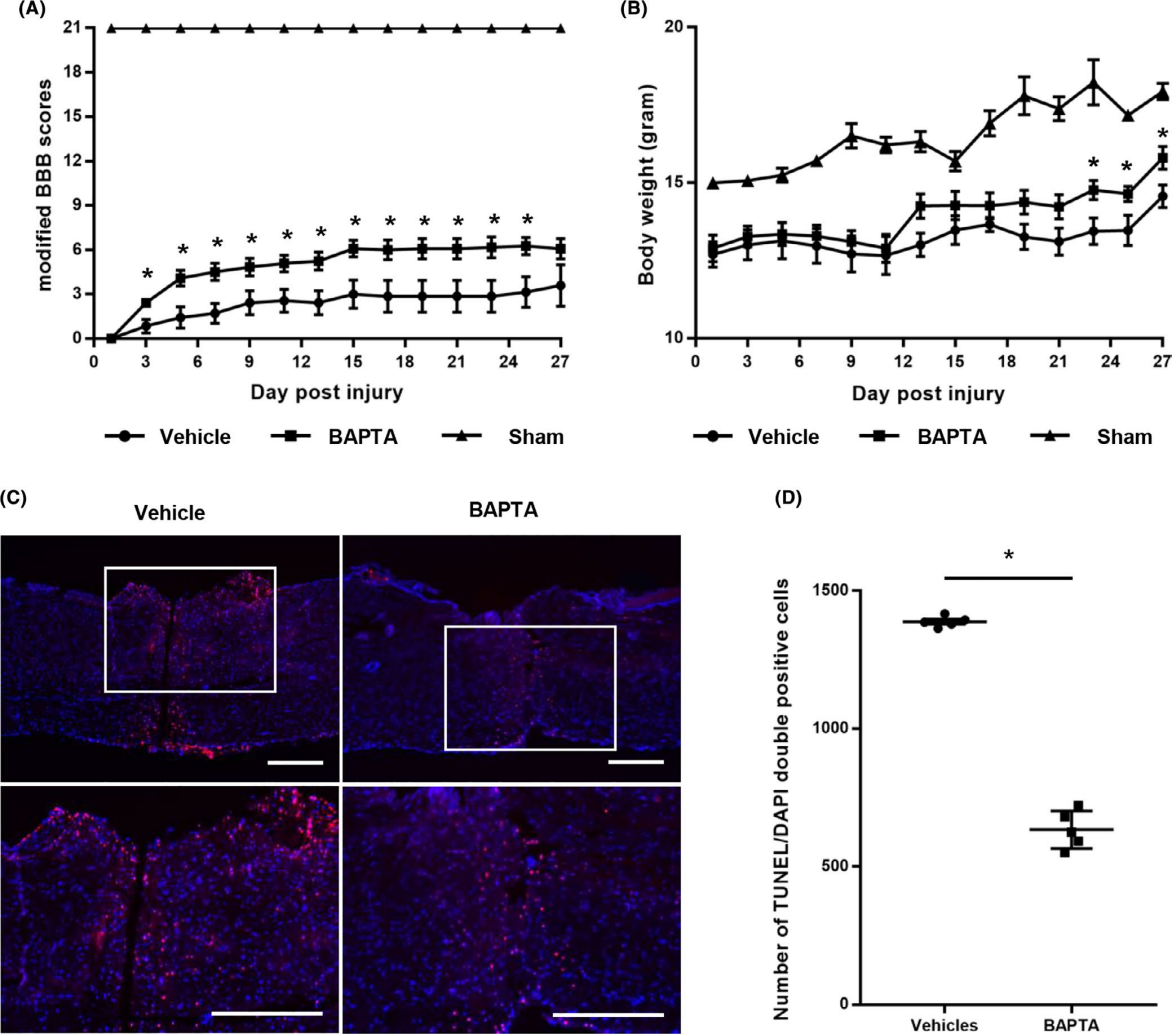
BAPTA inhibits apoptosis of cells in primary stage of SCI and improve locomotor recovery. (A) Changes in modified BBB scores after SCI. (B) Change in the body weight after SCI. (C) Representative TUNEL assay images of spinal cord injury model mice. Low‐magnification (upper panels) and high‐magnification (lower panels) IHC images showed significantly different number of TUNEL/DAPI double‐positive cells; bar = 500 μm. (D) Quantification of apoptotic cells by counting TUNEL/DAPI double‐positive cells. Statistical significance is shown as follows: *P < 0.05 (Mean ± SEM, n = 5‐7)

**FIGURE 2:**
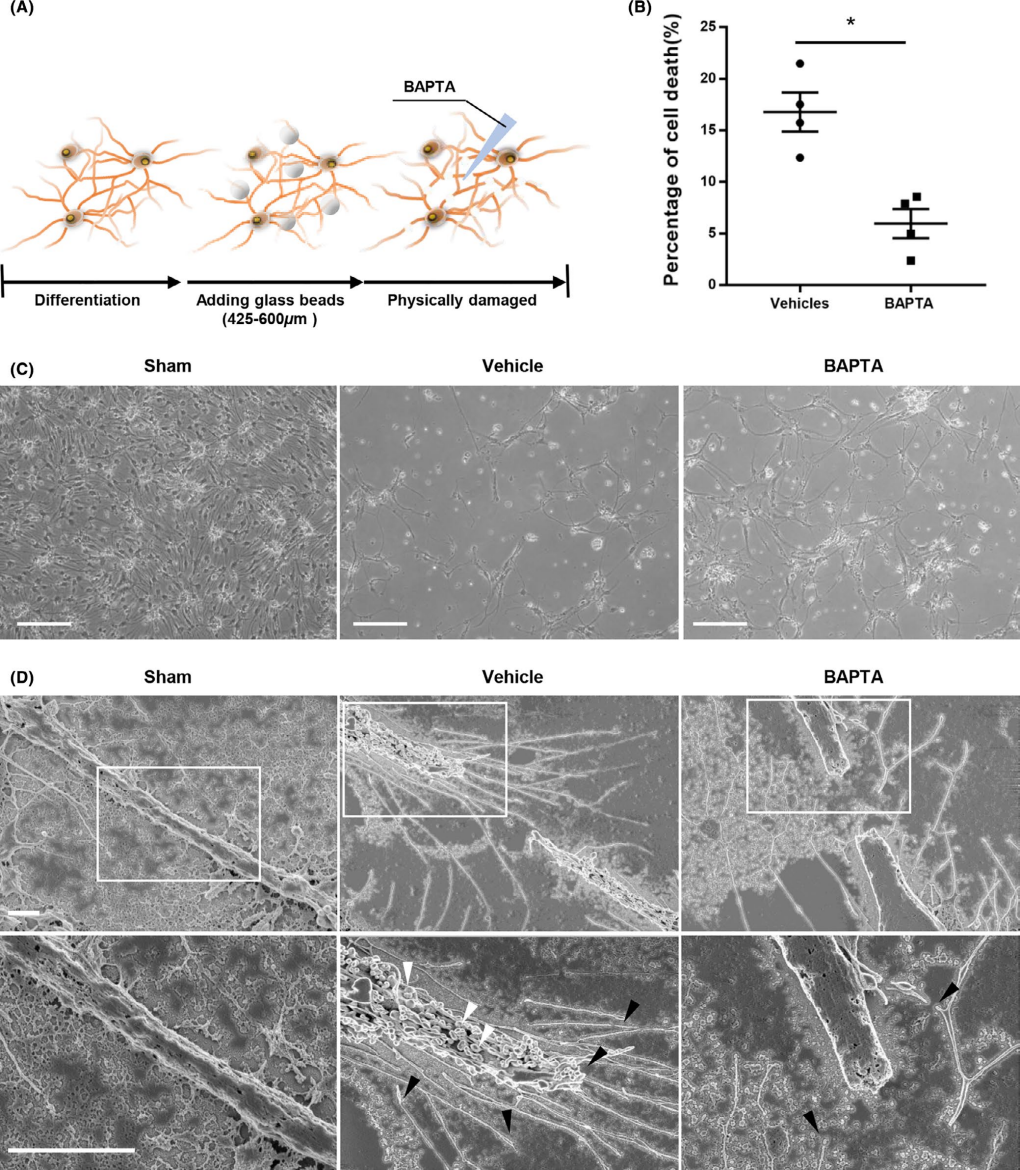
BAPTA relieved the death of neurons caused by physical damage. (A) Scheme of experiments. The process of generating in vitro model of SCI and experiments for testing the function of BAPTA. (B) Quantitative analysis of cell deaths. Dead cells were collected and determined by trypan blue staining and cell count. The graphs are representative of three independent experiments. Statistical significance is shown as follows: *P<0.05 (Mean ± SEM, n = 3). (C) Bright‐field microscopy images of floating dead cells. Sham shows neurons without damage. Vehicle controls showed the neurons treated DPBS after bead damaging. BAPTA means the neuron‐treated BAPTA after bead damaging; bar = 50 μm. (D) Low‐magnification (upper panels) and high‐magnification (lower panels) SEM images of damaged neurons. White box showed magnified field. Arrows indicated general marker of apoptosis such as broken and shrunk neurites (black arrows) and blebs (white arrows); bar = 1 μm

**FIGURE 3:**
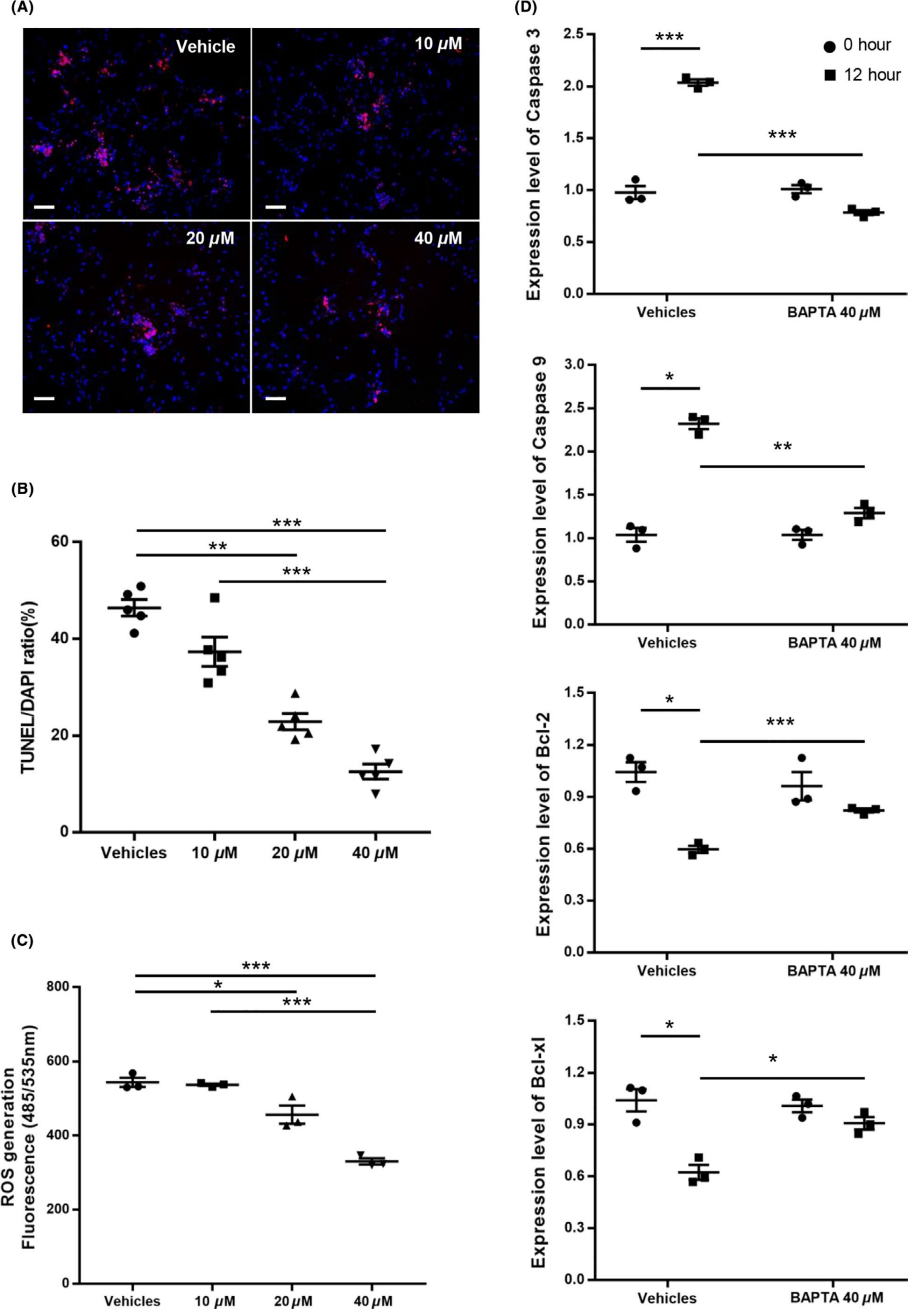
BAPTA inhibits ROS generation and neuronal apoptosis. (A) TUNEL assay images of different concentration of BAPTA‐treated group after bead damaging; bar = 50 μm. (B) Quantification of apoptotic neurons by counting TUNEL/DAPI double‐positive cells. (C) Accumulated ROS level detected by DCF‐DA. (D) Changes in apoptosis‐related gene expression level depend on time and condition. All the values were normalized to the value of the sample collected immediately after 30 minutes of BAPTA (or DPBS) treatment. Statistical significance is shown as follows: *P < 0.05, **P < 0.01, ***P < 0.001 (Mean ± SEM, n = 3)

**FIGURE 4:**
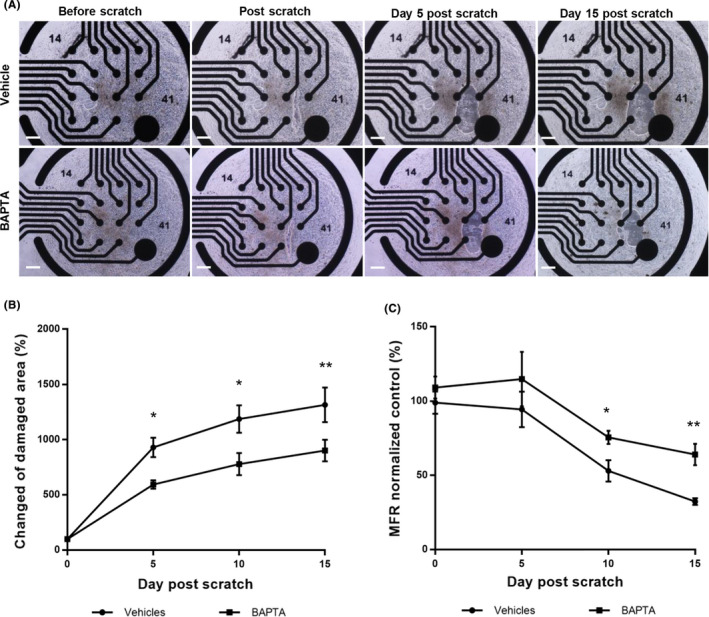
BAPTA relieves impairment of neuronal function after physical damaging. (A) Representative bright‐field microscopy images of neurons after scratch; bar = 50 μm. (B) Quantification of expanded damaged area for 15 days after scratching. (C) Quantification of decreasing MFR normalized by the MFR of day 0 for 15 days after scratching. Statistical significance is shown as follows: *P < 0.05, **P < 0.01, (Mean ± SEM, n = 4‐7)

All of the data were analyzed by D’Agostino‐Pearson omnibus normality test to assess normal distribution. Mann‐Whitney test was used for two experimental groups (Figure 1A,B,D, Figure 2B, Figure 3D, Figure 4B,C) in case the data do not exhibit normal distribution. If there were 3 or more experimental groups and did not exhibit normal distribution by D’Agostino‐Pearson omnibus normality test (Figure 3B, C), one‐way ANOVA (Kruskal‐wallies test) was used. GraphPad Prism 7.00 was used for analyzing whole data. Also, all the data were presented as a mean ± SEM and the statistical significance was determined at *P *≤ 0.05.

## 3 RESULTS

### 3.1 In vivo assessment

#### 3.1.1 Typical BAPTA improves locomotor recovery in vivo and inhibits neuronal apoptosis in the SCI model

The mBBB score increased significantly in both the BAPTA group and the vehicle control group for the first five days following SCI. However, the mBBB scores in the BAPTA group were significantly higher than the scores of the vehicle control from the 3rd day following SCI (Figure 1A). Body weight in the sham, vehicle, and BAPTA groups all increased sequentially over a month following SCI. The bodyweight of the BAPTA group was significantly higher than that of the vehicle control from the 23th day following SCI (Figure 1B).

Out of 23 mice, 15 mice were sacrificed for TUNEL staining. Twenty‐eight days following injury, TUNEL‐positive cellular nuclei appeared at the injured site. The majority of the TUNEL‐positive cells were scattered at the boundary and inside the spinal cord sections in both groups (Figure 1C and Figure S1A). However, the number of positive cells was significantly lower (1386.5 ± 10.6 vs 646 ± 120.2) in the BAPTA‐treatment group in comparison with the vehicle control group (Figure 1D).

### 3.2 In vitro model

#### 3.2.1 BAPTA relieves traumatic neuronal death in vitro

For evaluation the effect of BAPTA, differentiated neuron was damaged by glass beads (Figure 2A). The sham group evinced no detached cells. In the BAPTA‐treated group, 5.3% of the cells had detached from the plate, versus 14.5% in the vehicle control group (Figure 2B,C). Under SEM (Figure 2D), neurons in the vehicle control group (but not in sham controls) displayed apoptotic traits. Broken neurites were surrounded by a number of blebs caused by disruption of the cytoskeleton, and terminal parts of disconnected neurites were atrophied and shrunken. On the contrary, most of the neurites in the BAPTA‐treated group revealed a smooth surface without blebs or perforation.

#### 3.2.2 BAPTA inhibits ROS generation and neuronal apoptosis

The concentration ranges were set serially from 10 μM according to the previous study.^15^, ^16^ The 10 μM, 20 μM, and 40 μM of BAPTA were used in experiments. The number of TUNEL‐positive cells was significantly decreased in a dose‐dependent manner, and at 40 μM, BAPTA was maximally effective (Figure 3A,B). Consistent with Figure 3B, the ROS level on the DCF‐DA assay was significantly reduced and showed its lowest level at 40 μM, depending upon the BAPTA concentration (Figure 3C). The representative apoptosis marker expression levels, such as *Caspase 3* and *Caspase 9*, were increased over time in the group not treated with BAPTA. However, the expression level of *Caspase 3* and *Caspase 9* did not significantly change in the BAPTA‐treatment group. Eventually, following 12 hours of BAPTA treatment, the *Caspase 3* expression level was 2.7 times less as compared to the *Caspase 3* expression level in the vehicle control group. The *Caspase 9* expression level following 12 hours of BAPTA treatment was also 1.5 times less than the vehicle control group. In the case of antiapoptosis markers, such as *Bcl*‐*2* and *Bcl2 l1*, expression levels were both decreased in the vehicle control group following 12 hours, whereas the expression levels in the BAPTA‐treatment group were not significantly different. Finally, following 12 hours of BAPTA treatment, *Bcl*‐*2* and *Bcl*‐*xl* were expressed 1.4 and 1.5 times higher as compared to the vehicle control group (Figure 3D).

#### 3.2.3 BAPTA treatment relieves impairment of neuronal electrophysiology after physical damaging

The scratches expanded rapidly for 5 days following the initial scratching and then slowly expanded until day 15 (Figure 4A). From day 5, the expanded scratch area was significantly less extensive in the BAPTA‐treated group than in the DPBS group (Figure 4B). Initially, glass scratching did not result in electrical dysfunction, but from the day 5 through 15, overall MFR was dramatically decreased: the MFR in the vehicle control group decreased from 98.8% ±18.6% to 32.3% ±5.8%, versus from 109.1%±19.5 to 64.0%±19.0% in the BAPTA‐treated group. On day 15, the MFR of the BAPTA‐treated group was significantly lower than the vehicle control group (Figure 4C).

## 4 DISCUSSION

The present study is the first to confirm the effect of BAPTA as a neuroprotective agent following SCT, the most severe form of injury to the spinal cord. Unlike previous animal and clinical trials^15^ in which Ca++ antagonists were deployed orally and parenterally, we find that Ca++ antagonism can in fact protect the cells in the context of SCI when the agent is locally and immediately applied to the site of injury. BAPTA can inhibit neuronal apoptosis (neuron, astrocyte etc. in spinal cord) in the early stages of SCI, conserve electrophysiological integrity and ultimately lead to locomotor recovery after SCT in mice.

Local/immediate application (LIA) has been shown to be an effective strategy after SCT in other contexts. In particular, LIA of polyethylene glycol (PEG) reversed motor paralysis following SCT in rodents, canines, and primates.^29^ LIA has been theorized as a feasible strategy in the context of human SCI and is currently being tested in an ongoing clinical trial, with initial promising results.^30^ In particular, it has been proposed that the only feasible therapeutic approach to chronic SCI is removal of the most heavily damaged portion of the cord and replacement with healthy tissue.^31^ PEG primarily acts by reducing apoptosis of gray matter cells allowing them to resprout and reestablish communication across the treatment interface between the primary cord and the grafted tissue. In such a scenario, other chemicals with a similar profile to PEG can be explored, for possible combination treatment.

BAPTA, a high affinity Ca++ chelator, has long been recognized as neuroprotective in cell cultures. Wang et al^32^ described BAPTA’s potent Ca++ buffering properties on mechanically elicited intercellular Ca2+ waves in cell cultures, with other chelators such as tetrakis (2‐pyridylmethyl) ethylenediaminea (TPEN) less effective. As a consequence, neurodegeneration caused over 24 hr by 60 min of oxygen‐glucose deprivation (OGD) in cultured hippocampal slices and triggered largely by NMDA receptor activation was attenuated temporarily by pretreatment with BAPTA; protection by Ca2+ buffering originated presynaptically. In other words, enhancing neuronal Ca2+ buffering unequivocally attenuates or delays the onset of anoxic neurodegeneration, by attenuating excitotoxicity.^33^ BAPTA also mitigated neural injury in ALS models^15^ and following cortical stab injury.^16^

Our study shows for the first time that locally applied BAPTA immediately after in vivo SCT curbed apoptotic cell death and stifled ROS generation. Importantly, BAPTA‐AM promoted recovery of compound action potentials (CAP) after transection SCI in mice. No other study assessed BAPTA after traumatic SCI, and only one study examined the effects of BAPTA‐AM following hypoxic/reperfusion injury of spinal cord dorsal columns in rats.^34^

If confirmed, these data position BAPTA‐AM as a further chemical that might be transitioned into a SCI trial of spinal cord reconstruction, as currently enacted with other molecules.

## CONFLICT OF INTEREST

The authors declare no competing financial interests.

## Supporting information

Figure S1

Figure S2

Figure S3

## Data Availability

The data that support the findings of this study are available from the corresponding author upon reasonable request.
