# Supplementary figures and images for: BAPTA, a calcium chelator, neuroprotects injured neurons in vitro and promotes motor recovery after spinal cord transection in vivo
Source: CNS Neurosci Ther. 2021 May 4;27(8):919–29. doi: 10.1111/cns.13651 (PMC8265943; doi:10.1111/cns.13651)

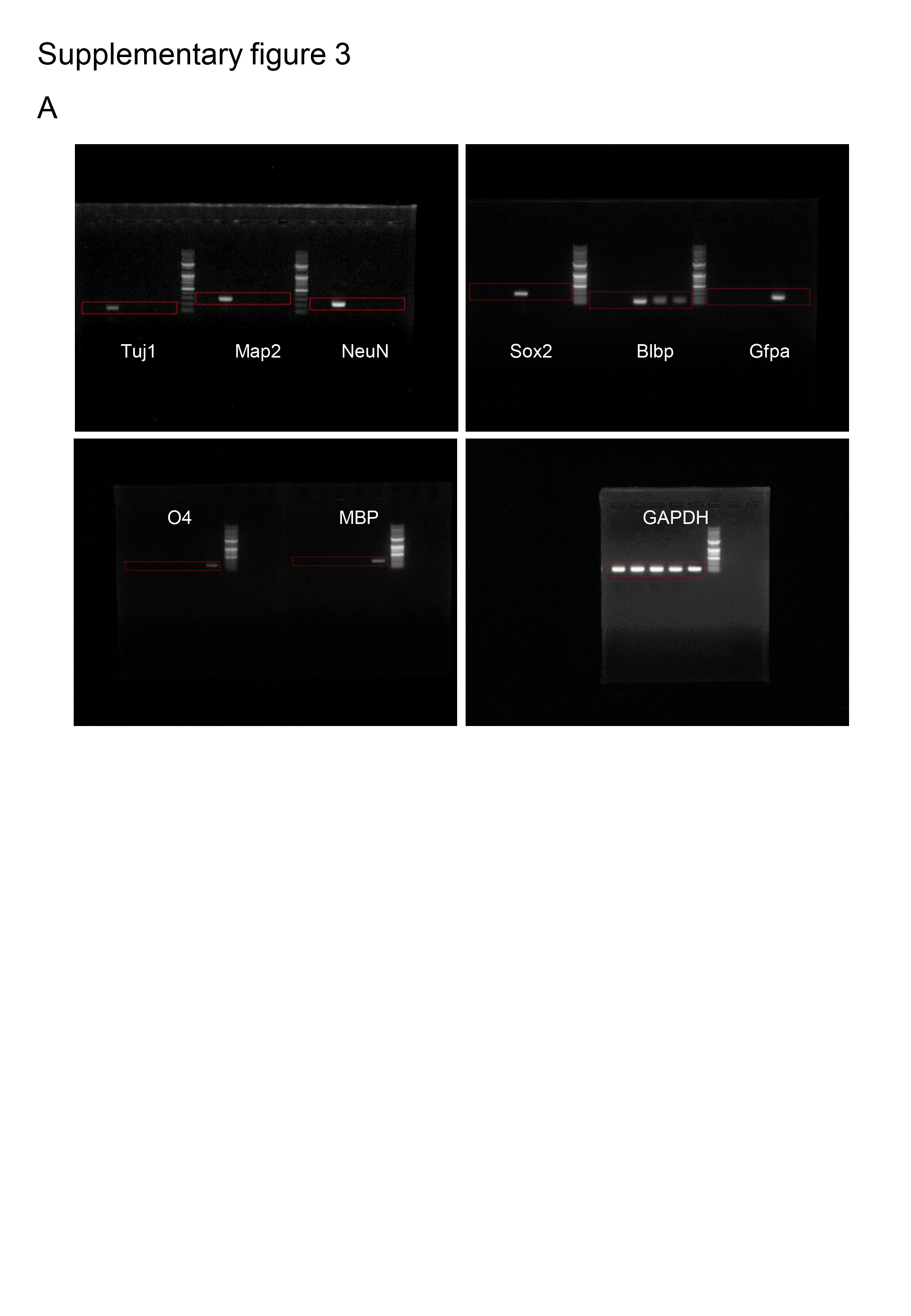

Supplement: Supplementary file 1 — Figure S1 [file CNS-27-919-s001.tif]

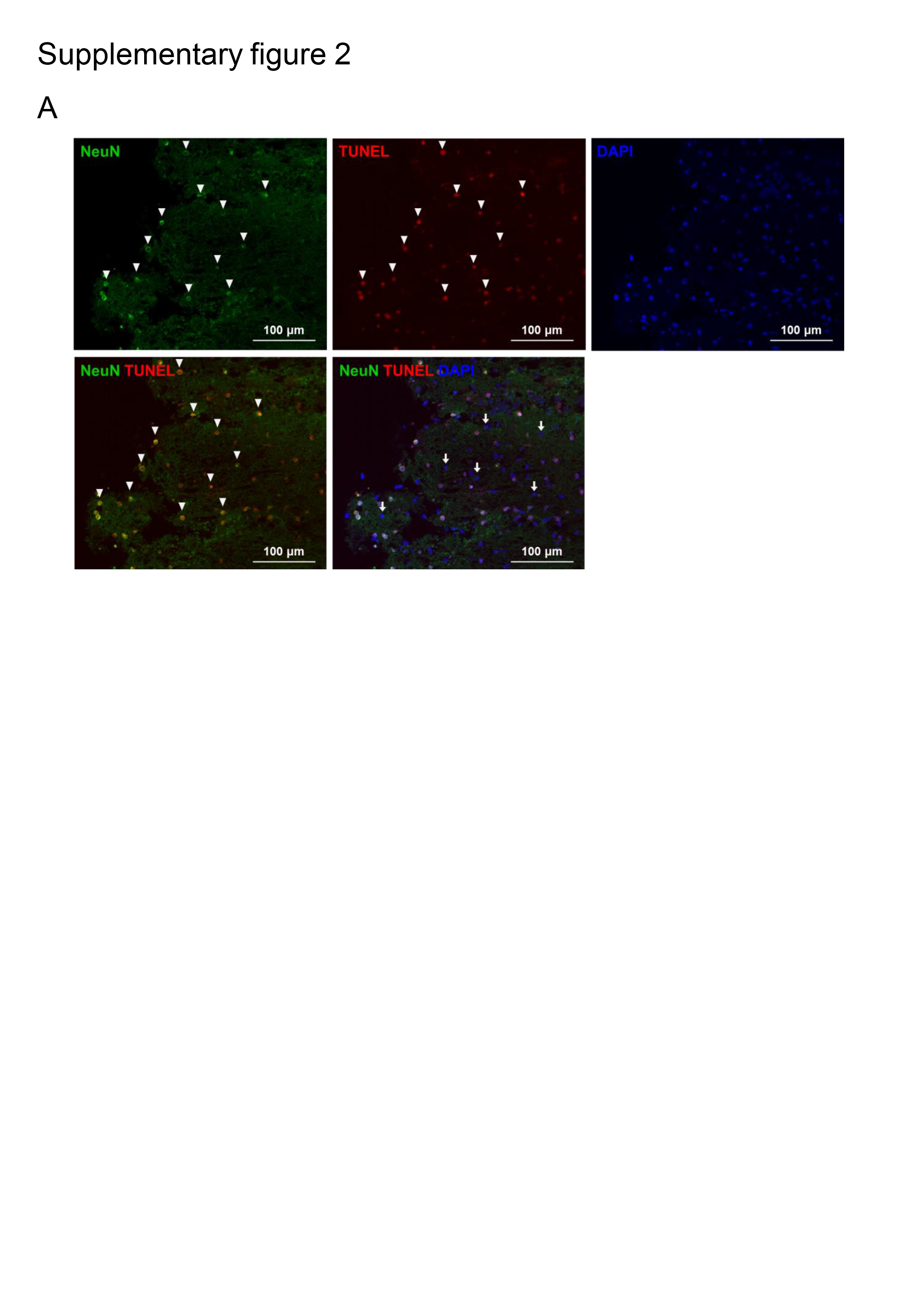

Supplement: Supplementary file 2 — Figure S2 [file CNS-27-919-s003.tif]
